# Lipid Oxidation Inhibitory Effects and Phenolic Composition of Aqueous Extracts from Medicinal Plants of Colombian Amazonia

**DOI:** 10.3390/ijms13055454

**Published:** 2012-05-04

**Authors:** Leandro J. Lizcano, María Viloria-Bernal, Francisca Vicente, Luis Angel Berrueta, Blanca Gallo, Magdalena Martínez-Cañamero, Maria Begoña Ruiz-Larrea, José Ignacio Ruiz-Sanz

**Affiliations:** 1 Department of Physiology, Medicine and Dentistry School, University of the Basque Country UPV/EHU, 48940-Leioa, Spain; E-Mails: lizcanomvz@gmail.com (L.J.L.); joseignacio.ruizs@ehu.es (J.I.R.-S.); 2 Department of Analytical Chemistry, Sciences and Technology School, University of the Basque Country UPV/EHU, 48940-Leioa, Spain; E-Mails: maria.viloria@ehu.es (M.V.-B.); francisca.vicente@ehu.es (F.V.); luisangel.berrueta@ehu.es (L.A.B.); blanca.gallo@ehu.es (B.G.); 3 Department of Health Sciences, Microbiology Area, Experimental Sciences School, University of Jaen, 23071-Jaen, Spain; E-Mail: canamero@ujaen.es

**Keywords:** polyphenols, lipid peroxidation, liver microsomes, HPLC-DAD-MS/MS, Amazonian plants

## Abstract

Diverse plants of ethnobotanic interest in Amazonia are commonly used in traditional medicine. We determined the antioxidant potential against lipid peroxidation, the antimicrobial activity, and the polyphenol composition of several Amazonian plants (*Brownea rosademonte*, *Piper glandulosissimum*, *Piper krukoffii*, *Piper putumayoense*, *Solanum grandiflorum*, and *Vismia baccifera*). Extracts from the plant leaf, bark, and stem were prepared as aqueous infusions, as used in folk medicine, and added to rat liver microsomes exposed to iron. The polyphenolic composition was detected by reverse-phase HPLC coupled to diode-array detector and MS/MS analysis. The antimicrobial activity was tested by the spot-on-a-lawn method against several indicator microorganisms. All the extracts inhibited lipid oxidation, except the *P. glandulosissimum* stem. The plant extracts exhibiting high antioxidant potential (*V. baccifera* and *B. rosademonte*) contained high levels of flavanols (particularly, catechin and epicatechin). By contrast, *S. grandiflorum* leaf, which exhibited very low antioxidant activity, was rich in hydroxycinnamic acids. None of the extracts showed antimicrobial activity. This study demonstrates for the first time the presence of bioactive polyphenolic compounds in several Amazonian plants, and highlights the importance of flavanols as major phenolic contributors to antioxidant activity.

## 1. Introduction

Medicinal plants are sources of therapeutic compounds. Thus, most of the actual drugs derive from plants, which are natural resources in indigenous communities. For instance, the alkaloids, atropine and scopolamine were isolated from plants of the *Solanum* genus, and are commonly used in medical applications as antispasmodic, sedative, and anticholinergic agents [1,2]. Among the natural effects of plant extracts, many actions, such as vasodilator, antimicrobial, sedative, anti-depressive, anti-pyretic, and anti-inflammatory are highlighted. There is an increasing interest in finding natural bioactive molecules from plants, in order to avoid side effects associated with synthetic drugs. The rainforest in Northwest Amazonia (Colombia, Ecuador, and Peru) represents a large area of ethnobotanical interest [3]. Historically, indigenous communities in these regions have used botanical resources with therapeutic activities [4,5]. In most of the cases, their use is limited to their intake as food after being cooked or as infusions, but other forms, such as maceration and its application to the skin, or vapour inhalation from infusions are also used. The *Brownea rosademonte* bark is used against snake venom, acting as an anticoagulant, also as a haemostatic against internal bleeding and haemorrhages, as well as against prolonged menstruation [6,7]. The plant species *Solanum grandiflorum* is widely used for its sedative, relaxant, and anti-spasmodic properties, and also in the treatment of skin infections [8,9]. Several *Vismia* species have been used against skin diseases, such as dermatitis, herpes, eczemas and wounds [10–12]. Cytotoxic and antiplasmodial [13,14], antiprotozoal [15], anticancer activity [16], HIV-inhibitory [17] and antimicrobial activities [18] were attributed to substances isolated from the *Vismia* species.

Many of the therapeutic actions of phytochemicals are ascribed to their biologically active polyphenol components, such as flavonoids and phenolic acids, which possess powerful antioxidant activities [19,20]. It is important for pharmacological purposes to screen, analyse, and identify these constituents.

The antioxidant activities, evaluated by the Trolox equivalent antioxidant activity (TEAC) and oxygen radical absorbance capacity (ORAC) assays, of different Amazonian plants prepared as aqueous infusions have been previously described, all the extracts showing different degrees of antioxidant activities. However, most of phytochemicals are multifunctional, and total antioxidant activity based solely on one property, such as their scavenging activity towards artificial radicals, provides no information on what lipid or other substrate is protected.

In the present work, we have assessed the lipid peroxidation inhibitory effects of aqueous extracts of six Amazonian plants, using rat liver microsomes as the lipid source. This lipid model system mimics the physiological target system to be protected. In addition, the phenolic composition of the extracts was characterised by reverse phase HPLC coupled to a diode array detector (DAD) and MS/MS analysis.

## 2. Results and Discussion

### 2.1. Inhibition of Lipid Peroxidation

**Figure 1:**
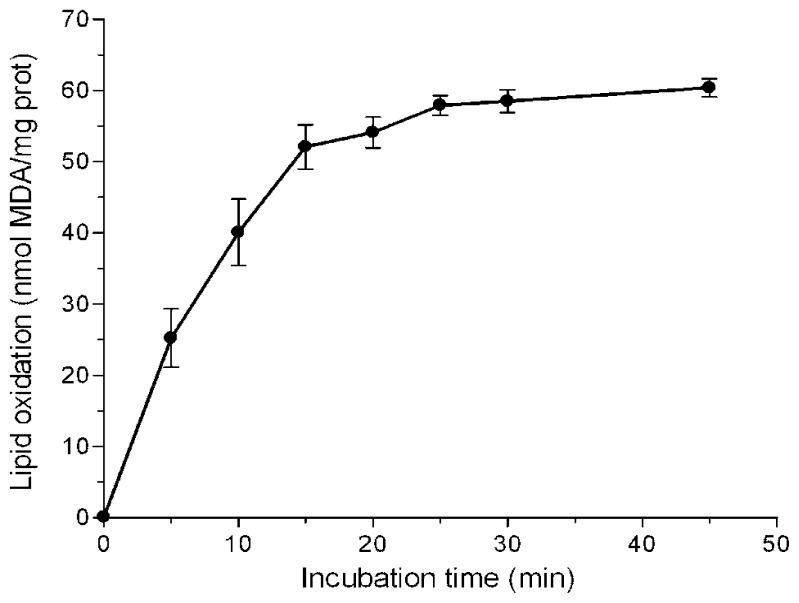
Time-course of iron-induced lipid peroxidation of rat liver microsomes. Microsomes (0.5 mg protein/mL) were incubated with 25 μM FeSO_4_ and 500 μM ascorbic acid in 10 mM KH_2_PO_4_, pH 7.4. Each value is the mean of 5 independent assays.

In this work six Amazonian species that had been shown to exert high *in vitro* antioxidant activities against hydrophilic radicals [21] were selected in order to analyse their protective effect against lipid peroxidation. The bark of *B. rosademonte* and leaves and stems of *P. glandulosissimum*, *P. krukoffii*, *P. putumayoense*, *S. grandiflorum* and *V. baccifera* were prepared as aqueous infusions, as are commonly used in folk medicine. Rat liver microsomes exposed to iron/ascorbate were chosen as an oxidative system because it is close to the *in vivo* situation where both an aqueous phase and a lipid phase are present. Figure 1 shows the time-course of thiobarbituric acid reactive substances (TBARS) production in liver microsomes without antioxidants.

**Figure 2:**
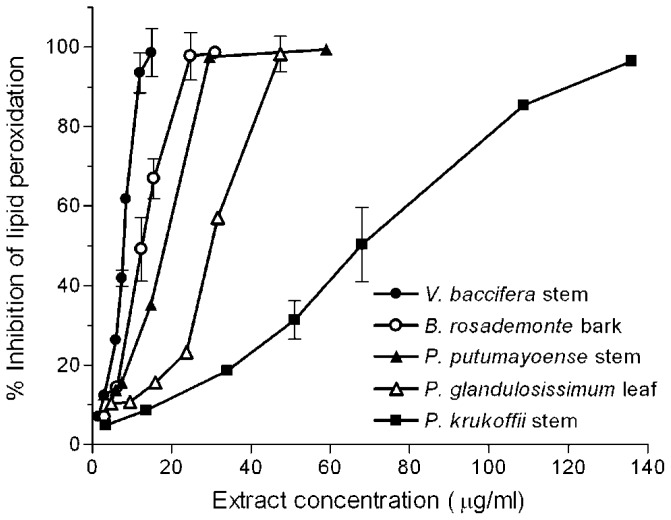
Inhibition of iron-induced microsomal lipid peroxidation by plant extracts. Microsomes (0.8 mg protein/mL) were incubated for 10 min with FeSO_4_/ascorbate (25 μM/500 μM) in the presence of increasing quantities of the indicated plant extracts in a 1 mL final volume. Each value is the mean of at least 3 independent assays.

With short incubation periods TBARS levels increased linearly with time, reaching maximum values at near 25 min. A 10 min incubation period was chosen to study the effects of the extracts. Different volumes of the water infusions were added to the microsome solutions, so that a final 20–80% inhibition could be detected. Figure 2 shows typical concentration-dependent inhibition curves exhibited by some of the assayed extracts.

The quantity of extract which inhibited control malondialdehyde (MDA) production by 50% (IC_50_) was determined from the curves. Results are summarised in Table 1. For comparative purposes, the IC_50_ for caffeic acid, catechin, and gallic acid was also measured.

The order of protection efficacies against lipid peroxidation of the reference antioxidants was catechin > gallic acid > caffeic acid. All the extracts exerted antioxidant effects against lipid peroxidation, except *P. glandulosissimum* stem, which showed no effect at the highest concentration used (240 μg/mL). *V. baccifera* extracts were the most potent antioxidants against lipid peroxidation, the IC_50_ values of the leaf (5.5 μg/mL) and stem (7.9 μg/mL) being even lower than that of gallic acid (8.8 μg/mL). *B. rosademonte* bark also showed a high inhibitory potential in a similar concentration range. *P. krukoffii* leaf and stem presented intermediate protective actions (IC_50_ near 70 μg/mL), while *S. grandiflorum* leaf was the least potent (near 100 μg/mL). Thus, using this lipid system, important differences in the behaviour exhibited by the extracts compared with that observed using aqueous free radicals can be highlighted. Lipid peroxidation is a free radical-mediated process, in which oxidative damage is propagated to polyunsaturated fatty acids. It involves lipid-derived radicals, such as alkoxyl and peroxyl radicals. The polarity features of the extract components is a key factor that confers their solubility and ability to access to the lipid phase where the lipoperoxidation process is taking place, thus influencing their capacity to break chain reactions. Antioxidant compounds with high partition coefficients are preferentially distributed to hydrophobic compartments, thus protecting lipids from free radical attack.

### 2.2. Antimicrobial Activity

The antimicrobial activity against several indicator strains (*L. monocytogenes*, *B. cereus*, *S. aureus*, *E. coli*, and *S. enterica*) was also tested. No antimicrobial activity was exhibited by any of the aqueous plant extracts (data not shown). Antimicrobial activity against Gram-negative and Gram-positive bacteria of extracts of the *Vismia* sepcies (*V. laurentii*) has been reported in the literature [18]. However, in contrast to our conditions, the authors used methanolic extracts to test their effectiveness on the pathogenic agents. In many cases, the water-soluble (aqueous) parts of an extract exhibit no activity or less than the water-insoluble ones [22,23]. To our knowledge this is the first report providing information regarding antimicrobial activity of aqueous extracts of the plants used in this study. The generalized lack of antimicrobial activity in these extracts supports their oral intake since it prevents them from jeopardizing the beneficial autochthonous intestinal microbiota.

### 2.3. Polyphenolic Composition by HPLC-DAD and MS/MS Analysis

**Figure 3:**
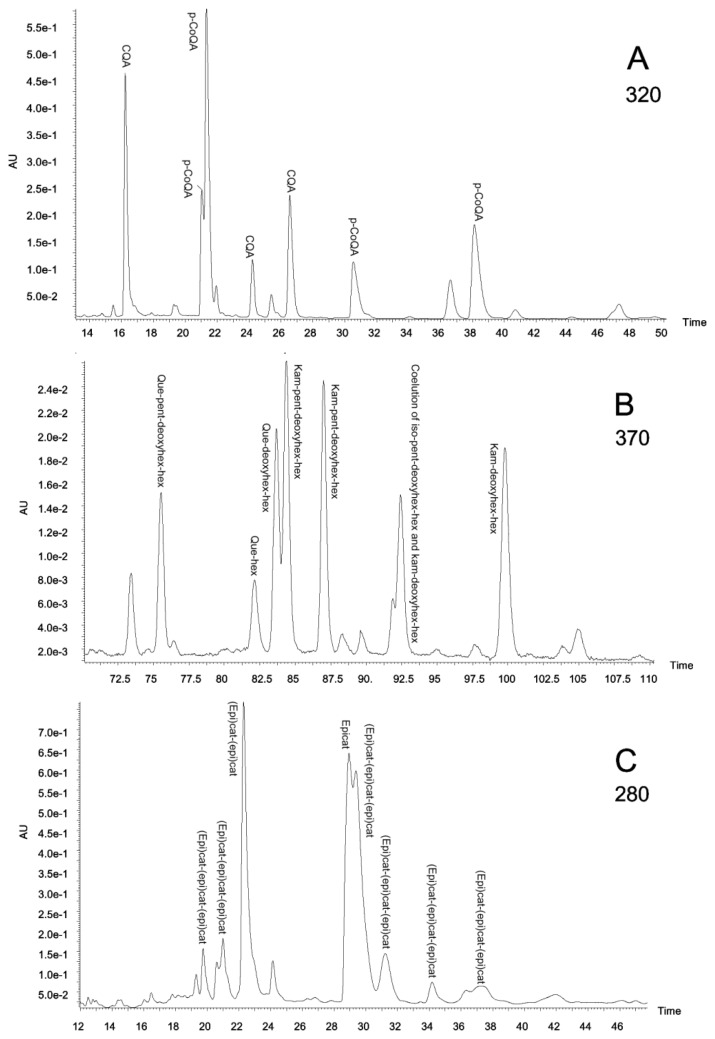
(**a**) *Piper glandulosissimum* leaf extract chromatogram at 320 nm; (**b**) *Piper glandulosissimum* leaf extract chromatogram at 370 nm; (**c**) *Vismia baccifera* leaf extract chromatogram at 280 nm. CQA, caffeoylquinic acids; deoxyhex, deoxyhexose; epicat, epicatechin; (epi)cat, epicatechin or catechin; hex, hexose; iso, isorhamnetin; kam, kaempferol; *p*-CoQA, *p*-coumaroylquinic acids; pent, pentose; que, quercetin.

The specific polyphenolic composition of the extracts was analysed by HPLC-DAD and MS/MS. To our knowledge, this is the first report showing the presence of an array of polyphenolic compounds in aqueous extracts of these Amazonian species used here. The presence of several families (flavanones, flavonols, hydroxycinnamic acids, hydroxybenzoic acids, flavones, coumarins and flavanols until a polymerization grade of three units) was searched by HPLC-DAD and MS/MS analysis. However, only three of these families (flavanols, flavonols and hydroxycinnamic acids) were found. Figure 3 shows typical chromatograms monitored at 280, 320 and 370 nm obtained for leaf extracts, where individual components of the three major polyphenol families (flavanols, hydroxycinnamic acids and flavonols) can be identified.

The HPLC retention times, the relative quantities of the individual peaks, and the identified compounds are shown in Table 2.

Interestingly, in *B. rosademonte* bark the only polyphenolic family detected was flavanol, including monomers and dimers. This phenolic species, also including trimers, was also the major component of *V. baccifera* stem, where only two quercetin derived flavonols were found. In contrast, quite a lot of hydroxycinnamic acids were detected in *S. grandiflorum*, these being the only polyphenolic family in the stem, whereas a few quercetin- and kaempferol-derived flavonols were also found in the leaf extract. Flavanols, flavonols and hydroxycinnamic acids were detected in *V. baccifera* leaf and *P. glandulosissimum* leaf and stem, although in *V. baccifera* leaf only a few hydroxycinnamic (caffeoylquinic) acids were observed.

Different plant organs normally have a different content of polyphenols, being higher in the leaf than in the stem. This is the case with *P. glandulosissimum*, where flavanols, flavonols and hydroxycinnamic acids were found in both parts, leaf and stem, but more compounds were detected in the leaf due to the higher polyphenolic concentration.

In *P. krukoffii* and *P. putumayoense* extracts no polyphenols of the type studied were found.

The plant extracts exhibiting high antioxidant potential against lipid peroxidation (*V. baccifera* and *B. rosademonte*) contained high levels of flavanols, according to their phenolic characterisation by HPLC; in particular, catechin and epicatechin were the most abundant flavanols (Tables 1 and 2). In the lipid model, the reference antioxidant catechin was found to be highly potent in preventing lipid oxidation, and about 12-fold more efficient than the hydroxycinnamic acid caffeic acid. This behaviour could explain the high antioxidant activity of the above extracts. By contrast, *S. grandiflorum* leaf, which exhibited very low antioxidant activity, was rich in hydroxycinnamic acids, in particular, ester forms of caffeic acid (caffeoylquinic and dicaffeoylquinic acids). It is interesting to note that the *S. grandiflorum* stem, in which HPLC analysis demonstrated lower quantities of polyphenols than the leaf, but a similar composition, was able to inhibit lipid peroxidation quite efficiently (IC_50_ = 24 μg/mL). These results indicate that additional phenolic species contained in the stem other than those analysed in this study (such as tannins) and/or other non-phenolic components (such as carotenoids and alkaloids) would contribute to the lipid peroxidation inhibitory activity of the stem.

## 3. Experimental Section

### 3.1. Chemicals and Standards

The phenolic standards, catechin and gallic acid were purchased from Sigma-Aldrich®, USA; gentisic acid, procyanidin B1, procyanidin B2, ferulic acid, sinapic acid, quercetin-3-*O*-galactoside, quercetin-3-*O*-glucofuranoside, querectin-3-*O*-glucopyranoside, quercetin-3-*O*-rhamnoside, kaempferol-3-*O*-glucoside, kaempferol-3-*O*-rutinoside, kaempferol-7-*O*-neohesperidoside, kaempferol-3-*O*-robinoside-7-*O*-rhamnoside, isorhamnetin-3-O-glucoside and isorhamnetin-3-*O*-rutinoside from Extrasynthèse (Genay, France); while siringic acid, (−)-epigallocatechin, (+)-catechin, (−)-epicatechin, 5′-caffeoylquinic acid, caffeic acid, p-coumaric acid and quercetin-3-*O*-rutinoside were provided by Sigma–Aldrich Chemie (Steinheim, Germany); caffeoyltartaric acid, apigenin-8-*C*-glucoside-4′-*O*-rhamnoside, quercetagetin and kaempferol-3-*O*-(*p*-coumaroyl)glucoside, by Chromadex (Santa Ana, CA, USA); and, quercetin dihydrated by Fluka Chemie (Steinheim, Germany).

Standard stock solutions were prepared in methanol. Dilutions from stock solutions were made in the initial mobile phase.

Methanol (Romil, Chemical Ltd., Heidelberg, Germany) was of HPLC grade. Water was purified on a Milli-Q system from Millipore (Bedford, MA, USA). Glacial acetic acid provided by Merck (Darmstadt, Germany), was of analytical quality. All solvents used were previously filtered through 0.45μm nylon membranes (Lida, Kenosha, WI, USA).

All other reagents used were from the highest purity available.

### 3.2. Plant Samples

Diverse plants of native Amazonian species with known therapeutic actions were collected from the Macagual Research Centre forest in Florencia, Caquetá (Colombia). The plants were selected on the basis of data from their traditional use in Colombian folk medicine. The plants were taxonomically identified by botanical experts and deposited in the Herbarium of the Botanical Garden of Amazonia University-HUAZ (Florencia, Colombia).

The plant samples were processed in the laboratory within a maximum of 24 h after harvesting. Otherwise, the material was stored under refrigeration at 4 °C.

### 3.3. Preparation of Plant Extracts

Plant extracts were obtained from infusions prepared as generally used in traditional medicine, as described before [21].

### 3.4. Inhibition of Lipid Peroxidation

Male Sprague Dawley rats (180–200 g) were used to isolate liver microsomes. The experimental use of animals and the particular procedure were approved by the Ethical Committee of Animal Welfare (CEBA) of the Institution. Rats were anesthetised and the liver was perfused with 0.9% NaCl to completely eliminate blood. Microsomes were obtained by differential centrifugation [24]. The microsomal pellet was resuspended in 150 mM Tris-HCl buffer, pH 7.4, and frozen in liquid N_2_ and stored at −80 °C. The protein content was determined as by Bradford [25], using bovine serum albumin as standard.

Incubations were performed in 150 mM Tris/HCl, pH 7.4, and liver microsomes (0.8 mg protein) in a final volume of 1 mL. Microsomes were preincubated with the different extracts for 10 min at 37 °C. Lipid oxidation was initiated by the addition of 200 μL FeSO_4_ (0.125 mM) and ascorbic acid (0.5 mM) in 10 mM KH_2_PO_4_. Incubations were stopped at selected times by the addition of 50 μL 100% trichloroacetic acid. Samples were maintained 10 min in ice and then centrifuged 10 min at 3200 × g. Supernatants were collected. Lipid oxidation was determined by the thiobarbituric acid (TBA) test [26]. Lipid peroxidation was expressed as nmol of malondialdehyde (MDA) per mg of protein, taking into account the molar extinction coefficient of MDA (ɛ = 156 mM^−1^·cm^−1^). Different dilutions of the plant extracts, showing a final percentage inhibition of 20–80%, were used. All the determinations were carried out in duplicate and the experiments were repeated at least three times. Fifty per cent inhibition of lipid peroxidation (IC_50_) was derived from the dose-response curves. The curves were adjusted by quadratic regression (*R*^2^ between 0.995 and 1). The lipid peroxidation half maximal inhibition values were expressed as μg of dry weight.

### 3.5. Antimicrobial Activity

The aqueous extracts were individually tested by the spot-on-a-lawn method against a panel of indicator microorganisms including *Listeria monocytogenes* (CECT 4032), *Bacillus cereus* (LWL1), *Staphylococcus aureus* (CECT 828), *Escherichia coli* (CECT 432), and *Salmonella enterica* (CECT 916). Indicator bacteria were inoculated (1%) into soft (0.75%) Brain Heart Infusion agar (BHI). This was used to overlay Brain Heart Infusion agar (BHA) plates. Then, the aqueous extracts were spotted on the surface of BHA previously inoculated with the indicator strains and after 12–18 hours incubation at 37 °C, the plates were examined for zones of inhibited growth around the spots.

### 3.6. HPLC-DAD and MS/MS Analysis

Chromatographic analyses were performed on a Waters (Milford, USA) Alliance 2695 coupled to a Waters 2996 diode array detector (DAD). A reversed-phase Phenomenex (Torrance, USA) Luna C18 column (150 mm × 4.6 mm i.d., particle size 3 μm) with a Waters Nova-Pack C18 guard column (10 mm × 3.9 mm i.d., 4 μm) was used. The flow rate and column temperature were set to 0.8 mL/min and 30 °C, respectively. A gradient program for general polyphenol analysis was employed: the eluents were acetic acid-water (0.5:99.5, v/v) (phase A) and methanol (phase B); initially 0% B for 2 min, a linear gradient to 15% B at 6 min, held isocratic until 12 min, linear gradient to 20% B at 17 min, 20% B constant until 35 min, linear up to 35% B at 90 min, 35% B constant until 136 min, and finally linear gradient to 0% B at 145 min.

A 50 μL volume of the plant extracts was injected. The chromatograms were monitored at 254 nm (to study hydroxybenzoic acids), 280 nm (for flavan-3-ols, condensed tannins, and flavanones), 320 nm (for hydroxycinnamic acids), 370 nm (for flavones, flavonols and coumarins); and complete spectral data were recorded in the range 200–600 nm each second.

Mass spectra were obtained on a Micromass (Milford, MA, USA) Quattro Micro triple quadrupole mass spectrometer equipped with a Z-spray ESI source coupled to the exit of DAD. A flow of 70 μL/min from the DAD eluent was directed to the ESI interface using a flow splitter. Nitrogen was used as desolvation gas, at 300 °C and a flow rate of 450 L/h, and no cone gas was used. A potential of 3.2 kV was used on the capillary for positive ion mode and 2.6 kV for negative ion mode. The source block temperature was held at 120 °C.

MS spectra, within the 50–1000 u *m*/*z* range, were performed at different cone voltages (CV): in the positive mode 15, 30 and 45 V; and in the negative mode 10, 20, 30 and 40 V. MS/MS product ions spectra were recorded using argon as collision gas at 1.5 × 10^−3^ mbar and different collision energies (CE) were assayed in the range 10–35 eV.

Individual polyphenols were classified within a polyphenolic family according to their UV-Vis spectra, whereas their identification was performed with the comparison of the MS/MS results with previous studies of standards fragmentation [27]. Peak area obtained from the DAD was used as relative quantitative data.

### 3.7. Statistical Analysis

Results were expressed as the mean ± standard deviation (SD). SPSS (version 16.0; SPSS Inc. Chicago, IL, 2007) statistical program was used for data analysis (Pearson’s correlation coefficient).

## 4. Conclusions

Our study has demonstrated, for the first time, the presence of bioactive polyphenolic compounds, such as catechin, epicatechin, kaempferol and quercetin, in six Amazonian plants. Most of the aqueous extracts of the plants also exhibited protection against lipid peroxidation. Flavanols, such as catechin and epicatechin, are suggested to largely contribute to the antioxidant activity of the plants, while ester forms of cinnamic acids would have minor contributions. Other components that have not yet been identified also seem to be involved in the lipid peroxidation inhibitory potential of the extracts. These plants may have important roles due to their antioxidant behaviour, and their oral consumption as aqueous infusions is supported for possible prevention of diseases associated with oxidative stress.

## Figures and Tables

**Table 1:** Lipid peroxidation half-inhibition values (IC_50_) of plant extracts and reference antioxidants.

		IC_50_		

Species	Plant Part	Concentration (μg/mL)	Total Phenols (μg GAE a/mL)	Total Flavonoids (μg CE b/mL)
*B. rosademonte*	bark	12.4	4.9	1.6
*P. glandulosissimum*	leaf	30.0	3.7	1.4
*P. glandulosissimum*	stem	ND	ND	ND
*P. krukoffii*	leaf	75.0	46.3	23.9
*P. krukoffii*	stem	68.0	34.0	17.2
*P. putumayoense*	leaf	17.9	8.7	4.5
*P. putumayoense*	stem	18.4	6.7	3.0
*S. grandiflorum*	leaf	97.7	22.4	5.5
*S. grandiflorum*	stem	24.1	4.5	2.0
*V. baccifera*	leaf	5.5	2.2	1.2
*V. baccifera*	stem	7.9	2.6	1.0
Caffeic acid		38.0		
Catechin		3.0		
Gallic acid		8.8		

ND: not detected at the highest concentration used (240 μg extract/mL);

a GAE, gallic acid equivalents;

b CE, catechin equivalents.

**Table 2:** Relative quantities (area units/mg of plant extract) of major polyphenols and sums of all polyphenols found for each phenolic family and plant species.

Major Polyphenols and Sums of the Different Families Found	Rt (min)	*B. rosademonte*	*P. glandulosissimum*	*P. glandulosissimum*	*S. grandiflorum*	*S. grandiflorum*	*V. baccifera*	*V. baccifera*
		Bark	Leaf	Stem	Leaf	Stem	Leaf	Stem
∑FA		171730	32296	7637	n.d.	n.d.	1971026	257339
(epi)cat-(epi)cat-(epi)cat	9.10	n.d.	9344	1369	n.d.	n.d.	n.d.	n.d.
(epi)cat-(epi)cat	14.85	15104	3094	n.d.	n.d.	n.d.	n.d.	n.d.
(epi)cat-(epi)cat	15.87	6731	4786	2630	n.d.	n.d.	n.d.	n.d.
(epi)cat-(epi)cat-(epi)cat	16.45	n.d.	n.d.	1685	n.d.	n.d.	n.d.	n.d.
(epi)cat-(epi)cat	17.58	12219	n.d.	n.d.	n.d.	n.d.	n.d.	n.d.
Cat	19.37	28346	9966	3225	n.d.	n.d.	-	-
(epi)cat-(epi)cat-(epi)cat	19.73	n.d.	n.d.	n.d.	n.d.	n.d.	50524	8592
(epi)cat-(epi)cat-(epi)cat	20.98	n.d.	n.d.	n.d.	n.d.	n.d.	82616	14036
(epi)cat-(epi)cat	22.30	45400	n.d.	n.d.	n.d.	n.d.	422618	64600
Epicat	28.93	48261	1285	413	n.d.	n.d.	463423	112225
(epi)cat-(epi)cat-(epi)cat	29.37	n.d.	n.d.	n.d.	n.d.	n.d.	523159	n.d.
(epi)cat-(epi)cat-(epi)cat	31.22	n.d.	n.d.	n.d.	n.d.	n.d.	129118	23137
(epi)cat-(epi)cat-(epi)cat	34.17	n.d.	n.d.	n.d.	n.d.	n.d.	41978	4828
(epi)cat-(epi)cat-(epi)cat	37.22	n.d.	n.d.	n.d.	n.d.	n.d.	83613	n.d.
(epi)cat-(epi)cat-(epi)cat	85.17	n.d.	n.d.	n.d.	n.d.	n.d.	n.d.	1627
∑FVL		n.d.	11232	967	12401	n.d.	106166	2696
Que-pent-deoxyhex-hex	75.53	n.d.	1045	165	n.d.	n.d.	n.d.	n.d.
Kam-deoxyhex-hex	76.18	n.d.	n.d.	n.d.	5605	n.d.	n.d	n.d.
Que-hex	82.28	n.d.	n.d.	n.d.	n.d.	n.d.	23930	n.d.
Que-deoxyhex-hex	83.73	n.d.	1521	344	n.d.	n.d.	6678	1788
Kam-pent-deoxyhex-hex	84.37	n.d.	2016	n.d.	n.d.	n.d.	n.d.	n.d.
Kam-pent-deoxyhex-hex	87.00	n.d.	1771	56	n.d.	n.d.	n.d.	n.d.
Coelution of iso-pent-deoxyhex-hex and kam-deoxyhex-hex	92.42	n.d.	1207	123^a^	n.d.	n.d.	n.d.	n.d.
Que-deoxyhex	96.18	n.d.	n.d.	n.d.	n.d.	n.d.	31258	908
Kam-hex	97.28	n.d.	n.d.	n.d.	n.d.	n.d.	22859	n.d.
Kam-deoxyhex-hex	99.78	n.d.	1877	n.d.	n.d.	n.d.	n.d.	n.d.
∑HCA		n.d.	111242	4323	1140885	565630	117394	n.d.
CQA	16.35	n.d.	18512	n.d.	110605	40783	n.d	n.d.
*p*-CoQA	21.03	n.d.	8420	n.d.	n.d.	n.d.	n.d.	n.d.
*p*-CoQA	21.32	n.d.	29335	1318	11566	n.d.	n.d	n.d.
CQA	24.13	n.d.	4568	n.d.	350237	239158	91738	n.d.
CQA	26.77	n.d.	12856	n.d.	112527	61954	n.d	n.d.
*p*-CoQA	30.53	n.d.	8222	389	n.d.	n.d.	n.d.	n.d.
*p*-CoQA	38.12	n.d.	17518	727	n.d.	n.d.	n.d	n.d.
diCQA	82.88	n.d.	n.d.	n.d.	221270	94436	n.d	n.d.
diCQA	84.60	n.d.	n.d.	n.d.	180674	49423	n.d	n.d.
diCQA	98.50	n.d.	n.d.	n.d.	110713	79875	n.d	n.d.

- not determined; n.d. not detected. In *P. glandulosisimum* stem only iso-pent-desoxihex-hex was found. CQA, caffeoylquinic acids; cat, catechin; deoxyhex, deoxyhexose; diCQA, dicaffeoylquinic acids; epicat, epicatechin; (epi)cat, epicatechin or catechin; FA, flavanols; FVL, flavonols; HCA, hydroxycinnamic acids; hex, hexose; iso, isorhamnetin; kam, kaempferol; *p*-CoQA, *p*-coumaroylquinic acids; pent, pentose; que, quercetin.
